# Prevalence of thyroid disorder in pregnant ladies among Maharashtrian women

**DOI:** 10.6026/9732063002001200

**Published:** 2024-10-31

**Authors:** Nikhila Cherukuri, Yamini Patil, Rajkumar P Patange

**Affiliations:** 1 Department of Obstetrics and Gynecology, Krishna Institute of Medical Sciences, Karad - 415110, Maharashtra, India

**Keywords:** Hypothyroidism, pregnant women, risk, complication, thyroid disorders

## Abstract

Thyroid disorders during pregnancy lead to maternal outcome and foetal outcome. Therefore, it is of interest to evaluate the prevalence of thyroid disorders in pregnancy and its correlation with maternal outcome and foetal outcome. Hence, 450 pregnant female patients were investigated on the basis of detailed history, clinical information and lab investigation to record maternal outcome and foetal outcome for Hyperthyroidism and hypothyroidism disorders. Data shows that those who had received treatment for hypothyroidism has less complications compared to those who had not received treatment. Thus, hypothyroidism warrants careful management to mitigate associated risks and complications.

## Background:

The second most common endocrine disorder in pregnant women is thyroid issues. In women, the prevalence of thyroid disorders is five to ten times greater than in males. Pregnancy leads to hypothyroidism due to several physiological changes. FT4 levels rise and TSH levels fall when HCG levels rise throughout the 1st trimester (Trim) [1]. According to a study, during pregnancy, the levels of Total T3 and Total T4 experience a 50% increase, which in turn results in a 50% rise in thyroxine binding globulin. In the 1st trim, serum TSH levels decrease, but they do not return to pre- pregnancy levels [1]. TSH levels also rise during the 2nd and 3rd trim. In pregnancy, overt hypothyroidism is observed in approximately 0.3-0.5% of cases, while subclinical hypothyroidism is seen in around 2-3%. Hyperthyroidism, on the other hand, is observed in about 0.1-0.4% of pregnancy. Autoimmune thyroid dysfunction (ATD) continues to be a prevalent issue during pregnancy. Women who are pregnant and have thyroid issues may face a range of challenges, including abortion (AB), premature birth, preeclampsia (P-EP), anemia, placental abruption and postpartum hemorrhage. Preterm births, stillbirths, IUGR and neonatal mortality are all examples of fetal complications. The effects of Disorders impact both the mother and the fetus [2]. The decreased level of TSH during the 1st trim is associated with a rise in HCG. This drop may have been caused by the modest stimulating actions of HCG on TSH receptors of the thyroid gland, which would have occurred owing to the molecular similarity between the α- subunit of HCG and TSH [3]. In early pregnancy, having high to normal FT4 levels can be linked to low-birth-weight babies and an increased risk of SGA newborns [4]. An untreated or inadequately treated woman with thyro-toxicity is at a higher risk for developing preeclampsia, experiencing AB, going into premature labor and giving birth to babies with low birth weight. Diagnosing hyperthyroidism during pregnancy can pose challenges as the physiological changes that occur during this time, such as fatigue, anxiety, elevated heart rate and basal metabolic rate, palpitations, heat sensitivity, warm and wet skin, hand tremors and systolic murmur, can complicate the process [5, 6]. In pregnant women with hyperthyroidism, there were notable findings such as more severe tachycardia and thyromegaly, along with exophthalmos and a lack of weight growth despite receiving appropriate nutrition. Based on the given information, it is clear that there is a reference to a source or citation [7]. Due to the unique and highly active state of pregnancy, thyroid disorders is often overlooked and not given proper attention in expectant mothers [8]. Therefore, it is of interest to report the prevalence of thyroid disorders in pregnancy and its correlation with maternal outcome and foetal outcome.

## Materials and Methods:

The current longitudinal prospective hospital- based observational study was conducted over 18 months, starting from June 2022 to November 2023 in the department of obstetrics of Krishna Hospital, Karad in total of 450 patients with the help of consecutive sampling to recruit the eligible pregnant women during their 1st antenatal visit, irrespective of GA. Data were collected from OPD, ward and labor room. Pre-determined proforma was used to record detailed history, clinical information which includes parity, mode of delivery (MOD) and its indication, GA at delivery, onset of labor, APGAR score at 1 and 5 min, birth weight of baby and neonatal intensive care unit admissions in the form of structured questionnaire. Other than this, lab investigations include routine ANC investigation, TSH level were measured at each trim. Additional tests, if indicated, were conducted based on clinical findings and standard antenatal care protocols and maternal outcome and foetal outcome were also recorded.

## Inclusion criteria:

[1] Patients assessed for TSH level along with other antenatal care (ANC) investigations.

[2] Those who were registered in other hospitals but coming for delivery to our hospital.

## Exclusion criteria:

Those women with confirmed thyroid disorders diagnose before pregnancy.

## Statistical analysis:

SPSS software was used to analyze the data. Additionally, descriptive statistics were used to summarize the data. Chi - square test and fisher's exact test were used to analyze categorical variables. T - Test and ANOVA were used for continuous variables.

## Result:

Table 1 shows that, out of total 450 pregnant women 28 (6.2%) had hypothyroidism and 2 (0.4%) had hyperthyroidism, 420 (93.3%) were Euthyroid. Table 2 shows that, in the 1st trim (N=308), the mean TSH level was 1.13 µIU/ml, with a SD of 1.26 µIU/ml. Moving to the 2nd trimester (N=408), the mean TSH increased to 1.99 µIU/ml, accompanied by a reduced SD of 0.81 µIU/ml. However, in the 3rd trimester (N=450), both the mean TSH level (3.58 µIU/ml) and the SD (1.73 µIU/ml) notably increased. Table 2 shows that, among women aged ≤ 20 years, there were no reported cases of hyperthyroidism and 3 (10.7%) case were reported hypothyroidism and 6.7% exhibiting normal thyroid function (TF). In the 21 - 30 years age group, hyperthyroidism was present in 2 case (100.0%), hypothyroidism in 18 cases (64.3%) and normal TF in 304 cases (72.4%). For women aged ≥ 31 to 40 years, hypothyroidism was reported in 7 cases (25.0%) and normal TF in 88 (21.0%). Therefore, there was no statistically significant association was observed between the age of mother at the time of registration and type of thyroid disorders as the p value was 0.645. Table 4 shows that, in primipara women (PM-W) group, 2 (100.0%) of hyperthyroidism cases, 11 (39.3%) of hypothyroidism cases and 188 (44.8%) of normal thyroid (NT) cases were observed, among 201 cases. In multipara women (MT-W) group, there were no cases of hyperthyroidism, while 17 (60.7%) had hypothyroidism and 232 (55.2%) found normal TF out of 249 cases. Therefore, not significant difference as the P value was 0.403. Table 5 shows that, among RW, 100.0% of hypothyroidism cases, 71.4% of hypothyroidism cases and 68.6% of NT cases were observed. For ROW, there were no cases of hyperthyroidism, 10.7% had HT and 29.5% had NTF. For URW, 17.9% had hypothyroidism and only 8 (1.9%) exhibited NTF. Therefore, we found not significant difference between the 2 variables as the p value was 0.142. Table 6 shows that, among all cases in the 1st trimester, 8 (28.6%) had hypothyroidism and 300 (71.4%) exhibited normal thyroid function. In the 2nd trimester, hyperthyroidism was present in 2 (100.0%) of cases, 18 (64.3%) had hypothyroidism and 76 (18.1%) showed normal thyroid group. All cases in the 3rd trimester, 2 (7.1%) were belong to hypothyroidism and normal thyroid function with 44 (10.5%). The P value of 0.003 indicates that gestational age at the time of registration was significantly higher in hypothyroidism compared to euthyriodism. Table 7 shows that, in cases where diagnosis occurred before 10 weeks of GA, 8 (28.6%) were diagnosed with HT and not any case was observed in hyperthyroidism group. For diagnoses occurring at 10 weeks or later, 2 (100.0%) of cases involved hyperthyroidism and 20 (71.4%) had HT. Therefore, found non-significant difference between GA at the time of diagnosis of thyroid disorders and type of thyroid as the p value was 0.487. Table 8 shows that, among cases with irregular MH, none had hyperthyroidism, 10 (35.7%) were diagnosed with HT and 28 (6.7%) showed NTF. Cases with regular MH, 2 (100.0%) had hyperthyroidism, 18 (64.3%) were diagnosed with HT and 392 (93.3%) exhibited NTF. There was statistically significance difference observed between past menstrual history and thyroid disorder group. (P= <0.001) this indicated that irregular menstrual bleeding is significantly higher in hypothyroid cases compared to euthyroid. Table 9 shows that, hyperthyroidism is associated with higher frequencies of complications such as pregnancy-induced hyperthyroidism (PIH) 4 (14.29%), preterm birth 7 (25.0%) and intrauterine growth restriction (IUGR) 2 (7.14%). Conversely, women with NTF exhibit fewer complications, with only 133 (29.6%) experiencing any adverse outcome compared to 20 (71.4%) among those with HT. In the normal group, such problems were GDM 24 (5.7%), Oligohydramnios 20 (4.8%), Preterm 20 (4.8%), PIH 16 (3.8%), IUGR 12 (2.9%), LBW 12 (2.9%), IUFD 8 (1.9%) and FSB 4 (1%). In hyperthyroidism group there was not any complication observed. Table 10 shows that, among women with hyperthyroidism, 100.0% underwent normal VD, while none required LSCS. Conversely, among those with HT, 16 (57.1%) had cesarean sections (CS), with 12 (42.9%) cases of normal VD. For women with NTF, 188 (44.8%) had normal VD and 232 (55.2%) underwent LSCS. Overall, CS was higher among women with hypothyroidism compared to hyperthyroidism and NTF. Therefore found a significant association as the p value was 0.020. Table 11 shows that, in hyperthyroidism group, 100.0% had an APGAR score of 7 or more. In hypothyroidism group, 8 (28.6%) scored 7 or more, while 20 (71.4%) scored between 4 to 6. Among NTF group, 328 (78.1%) had an APGAR score of 7 or more, with 64 (15.2%) scoring between 4 to 6 and 28 (6.7%) scoring less than 4. Therefore, found significant difference as the p value was 0.001. Table 12 shows that, among infants born to mothers with HT, 100.0% had an APGAR score of 7 or more. In contrast, hypothyroidism group, 20 (71.4%) scored 7 or more, while 8 (28.6%) scored between 4 to 6. For infants born to mothers with NTF, 364 (86.7%) had an APGAR score of 7 or more, with 32 (7.6%) scoring between 4 to 6 and 24 (5.7%) scoring less than 4. Thus, found significant difference as the p value was 0.001. Table 13 shows that, among women with hyperthyroidism, 100.0% did not require NICU admission, while among those with HT, 20 (71.4%) did not require NICU admission and for women with NTF 336 (80.0%) did not require NICU admission. These findings suggest that maternal hypothyroidism may be associated with a slightly higher NICU admission rate compared to NTF. Therefore, found non- significant difference as the p value was 0.586. Table 14 shows that, among those with hyperthyroidism, 100.0% received treatment, while among those with HT, 20 (71.4%) received treatment and 8 (28.6%) not received treatment. Overall, 73.3% of mothers received treatment for thyroid disorders, with the remaining 26.7% not receiving treatment. Table 15 shows that, a total of 20 cases with hypothyroid had received for HT, out of which in 6 (26.6%) cases has one other complications. In those who had not received treatment (n=8), 5 cases (78.1%) had developed complications. This difference was statistically significant (p<0.01) which indicates that those who had received treatment for hypothyroidism has less complications compared to those who had not received treatment.

## Discussion:

Thyroid adaptations are readily tolerated in an iodide-rich location because there is sufficient iodide stored inside the thyroid; these physiological adaptations cause pregnancy to vary significantly [9]. Studies have shown that, detecting and treating hypothyroidism early can help minimize potential risks for both the mother and the baby during pregnancy, as the treatment for this condition is relatively straightforward. In pregnant women, subclinical thyroid dysfunction is present in approximately 10% of cases, while over thyroid disorders occurs in about 2-3% of cases. In addition, it is estimated that the rate of autoimmunity falls between 5 and 10% [10, 11]. Maternal complications include miscarriage, anemia, preeclampsia, gestational age hyperthyroidism, placental abruption, premature birth, higher rates of caesarean section and postpartum hemorrhage. Delivery procedures have the potential to harm the fetal-pituitary-thyroid axis, leading to thyroid disorders, preterm delivery, low birth weight, respiratory issues, perinatal morbidity and mortality, increased hospitalization in the neonatal intensive care unit (NICU) and cognitive impairments. The development of the fetal brain is contingent upon the presence of thyroid hormone. Untreated congenital hypothyroidism leads to severe cognitive and developmental impairments. Offspring of mothers with hypothyroidism often have a lower intelligence quotient (IQ) compared to offspring of mothers without this condition [12]. Dhanwal *et al.* has studied the prevalence of hypothyroidism among women in 11 cities and 9 states of India. The incidence appears to be higher in India, in comparison with other countries [13]. Sahu *et al.* recorded 11.05% prevalence of hypothyroidism [14]. While Ajmani *et al.* [15] noticed 13.25% prevalence among the pregnant women of Delhi. Justin and Johnson *et al.* on the other had reported 10.54% hypothyroidism in Kerala, India [16]. Pahwa and Mangat *et al.* reported thyroid disorders in 10% of the pregnant women. Such a difference in the prevalence rate could be due to genetic variation in population [17]. Stagnaro-Green *et al.* reported 0.5 and 0.4% respectively in subclinical and overt hypothyroidism cases [18]. In present study 1st trim (N=308), the mean TSH level was 1.13 µIU/ml, with a SD of 1.26 µIU/ml. Moving to the 2nd trim (N=408), the mean TSH increased to 1.99 µIU/ml, accompanied by a reduced SD of 0.81 µIU/ml. However, in the 3rd trim (N=450), both the mean TSH level (3.58 µIU/ml) and the SD (1.73 µIU/ml) notably increased. In the study of Mahadik *et al.* found women with subclinical HT, overt HT and subclinical hyperthyroidism had mean serum TSH levels of 8.02±1.25 mIU/ml, 11.92 ± 5.34mIU/ml and 0.07±0.03mIU/ml, respectively [19]. Studies have shown that, women with subclinical HT, overt HT and subclinical hyperthyroidism had mean serum fT3 values of 2.92±0.454 pg/ml, 1.58±1.43 pg/ml and 4.16±0.40 pg/ml, respectively. Single nucleotide polymorphisms related with TSH, FT4, hyperthyroidism, HT and TPOAb were discovered from the most recent GWAS research [20, 21]. In present study, in hyperthyroidism group, 100.0% had an APGAR score of 7 or more. In hypothyroidism group, 8 (28.6%) scored 7 or more, while 20 (71.4%) scored between 4 to 6. Among NTF group, 328 (78.1%) had an APGAR score of 7 or more, with 64 (15.2%) scoring between 4 to 6 and 28 (6.7%) scoring less than 4. The significant P value of less than 0.001 indicates a strong association between hypothyroidism and lower APGAR scores compared to NTF. In addition to above, among infants born to mothers with hyperthyroidism, 100.0% had an APGAR score of 7 or more. In contrast, hypothyroidism group, 20 (71.4%) scored 7 or more, while 8 (28.6%) scored between 4 and 6. For infants born to mothers with NTF, 364 (86.7%) had an APGAR score of 7 or more, with 32 (7.6%) scoring between 4 to 6 and 24 (5.7%) scoring less than 4. The P value of less than 0.001 indicates a significant association between study groups and lower APGAR scores compared to NTF at 5 minutes. Moreover, overall, 73.3% of mothers received treatment for thyroid disorders with the remaining 26.7% not receiving treatment. Total 20 cases with hypothyroid had received for HT, out of which in 6 (26.6%) cases has one other complications. In those who had not received treatment (n=8), 5 cases (78.1%) had developed complications. This difference was statistically significant (p<0.01) which indicates that those who had received treatment for hypothyroidism has less complications compared to those who had not received treatment. Studies have shown that, preeclampsia was found in 13.6 percent of women with Sub-clinical hypothyroidism and 14.7 % of women with overt hypothyroidism [22, 23]. Increased rate of cesarean delivery is another outcome, observed in 26.7% (p = 0.012) of women with HT. Other authors have reported rates of cesarean delivery of 22.9% in women with hypothyroidism [22]. A study concluded that preeclampsia and rarely maternal heart failure have been linked to untreated or inadequately managed overt maternal hyperthyroidism during pregnancy [24]. During pregnancy, hyperthyroidism has been linked to fetal complications such as spontaneous abortion, premature delivery, IUGR and stillbirth [25]. The prevalence of thyroid disorders during pregnancy was observed to be 33.9%, with hypothyroidism occurring more frequently at 31.6% compared to hyperthyroidism, which was noted at 2.3%. They identified a notable correlation between thyroid disorders and feto-maternal complications. Thus they concluded that the adverse neonatal outcomes included low and very low birth weight, low Apgar scores, respiratory distress syndrome and meconium aspiration syndrome [26]. Even among rural populations, there is a high prevalence of thyroid dysfunction during pregnancy. Subclinical hypothyroidism is the most common of them. Early detection of thyroid dysfunction and prompt treatment are essential because maternal thyroid dysfunction significantly affects maternal and fetal outcomes. Due to the high prevalence of undiagnosed thyroid dysfunction in countries like India, universal screening of pregnant women with Sr. TSH during the first trimester should be emphasized. However, early diagnosis of thyroid dysfunctions followed by treatment during pregnancy improves the outcome [27].

## Conclusion:

Maternal outcomes indicated higher rates of Lower Segment Cesarean Section (LSCS) and its complications such as pregnancy-induced hyperthyroidism, preterm birth and intrauterine growth restriction in hypothyroid pregnancies, while hyperthyroid pregnancies had no significant complications and resulted in normal deliveries. Infants born to hyperthyroid mothers had excellent APGAR scores with no Neonatal intensive care unit admissions, whereas those born to hypothyroid and euthyroid mothers had varied outcomes. Thus, the importance of early and regular thyroid screening during pregnancy due to the significant impact of thyroid disorders on maternal and neonatal health is highlighted.

## Figures and Tables

**Table 1:** Women ACC TO TD

**Thyroid Status**	**Frequency**	**Percent**
Normal (Euthyroid)	420	93.30%
Hyperthyroidism(HYT)	2	0.40%
Hypothyroidism(HT)	28	6.20%
Total	450	100.00%

**Table 2:** TSH Value

**TSH measurement at the time of investigation**	**Mean (µ IU/ml)**	**SD**
1st Trimester (N=308)	1.13	1.26
2nd Trimester (N=408)	1.99	0.81
3rd Trimester (N=450)	3.58	1.73

**Table 3:** Age of women

**Age of Mother at the time of registration**	**Hyperthyroidism**		**Hypothyroidism**		**Normal**		**Total**	
	**Cases**	**%**	**Cases**	**%**	**Cases**	**%**	**Cases**	**%**
≤ 20 years	0	0.00%	3	10.70%	28	6.70%	31	6.90%
21 - 30 years	2	100%	18	64.30%	304	72.40%	324	72%
≥31 to 40 years	0	0%	7	25.00%	88	21%	95	21.10%
Total	2	100%	28	100%	420	100%	450	100%

**Table 4:** Women parity & TD

**Parity**	**Hyperthyroidism**		**Hypothyroidism**		**Normal**		**Total**	
	**Cases**	**%**	**Cases**	**%**	**Cases**	**%**	**Cases**	**%**
Primipara	2	100.00%	11	39.30%	188	44.80%	201	44.70%
Multipara	0	0.00%	17	60.70%	232	55.20%	249	55.30%
Total	2	100%	28	100%	420	100%	450	100%

**Table 5:** Registration status & TD

**Registration Status**	**Hyperthyroidism**		**Hypothyroidism**		**Normal**		**Total**	
	**Cases**	**%**	**Cases**	**%**	**Cases**	**%**	**Cases**	**%**
Registered(R)	2	100%	20	71.40%	288	68.60%	310	68.90%
Registered outside(RO)	0	0.00%	3	10.70%	124	29.50%	127	28.20%
Unregistered(UR)	0	0.00%	5	17.90%	8	1.90%	13	2.90%
Total	2	100%	28	100%	420	100%	450	100%

**Table 6:** GA at time of R & TD

**Gestational Age at the Time of**	**Hyperthyroidism**		**Hypothyroidism**		**Normal**		**Total**	
**registration**	**Cases**	**%**	**Cases**	**%**	**Cases**	**%**	**Cases**	**%**
1st Trimester (up to 13 weeks)	0	0%	8	28.60%	300	71.40%	308	68.40%
2nd trimester (14 to 28th week)	2	100%	18	64.30%	76	18.10%	96	21.30%
3rd Trimester (Above 28th week)	0	0%	2	7.10%	44	10.50%	46	10.20%
Total	2	100%	28	100%	420	100%	450	100%

**Table 7:** GA at time of diagnosis of TD

**Gestational Age at the time of diagnosis of thyroid disorder**	**Hyperthyroidism**		**Hypothyroidism**		**Total**	
	**Cases**	**%**	**Cases**	**%**	**Cases**	**%**	
<10 weeks	0	0.00%	8	28.60%	8	26.70%
≥10 weeks	2	100.00%	20	71.40%	22	73.30%
Total	2	100.00%	28	100.00%	30	100.00%

**Table 8:** PMH & TD

**Past Menstrual History(PMH)**	**Hyperthyroidism**		**Hypothyroidism**		**Normal**		**Total**	
	**Cases**	**%**	**Cases**	**%**	**Cases**	**%**	**Cases**	**%**
Irregular	0	0.00%	10	35.70%	28	6.70%	38	8.40%
Regular	2	100%	18	64.30%	392	93.30%	412	91.60%
Total	2	100%	28	100%	420	100%	450	100.00%

**Table 9:** MO & FO outcome

**Maternal**	**Hyperthyroidism**		**Hypothyroidism**		**Normal**		**Total**	
**Foetal outcome**	**Cases**	**%**	**Cases**	**%**	**Cases**	**%**	**Cases**	**%**
GDM	0	0.00%	2	7.14%	24	5.70%	26	5.30%
PIH	0	0.00%	4	14.29%	16	3.80%	20	5.30%
Oligohydramnios	0	0.00%	4	14.29%	20	4.80%	24	4.40%
Preterm	0	0.00%	7	25.00%	20	4.80%	27	6.20%
IUGR	0	0.00%	2	7.14%	12	2.90%	14	4.40%
IUFD	0	0.00%	0	0.00%	8	1.90%	8	1.80%
FSB	0	0.00%	0	0.00%	4	1.00%	4	0.90%
LBW	0	0.00%	6	21.43%	12	2.90%	18	2.70%
Spontaneous abortion	0	0.00%	0	0.00%	0	0.00%	0	0.00%
Any Complication	0	0.00%	11	39.30%	122	32.05%	133	29.60%
No Complication	2	100.00%	17	60.70%	298	69.50%	317	70.40%

**Table 10:** Type of delivery & TD

**Type of Delivery**	**Hyperthyroidism**		**Hypothyroidism**		**Normal**		**Total**	
	**Cases**	**%**	**Cases**	**%**	**Cases**	**%**	**Cases**	**%**
Normal VD	2	100%	16	57.10%	188	44.80%	206	45.80%
LSCS	0	0.00%	12	42.90%	232	55.20%	244	54.20%
Total	2	100%	28	100%	420	100%	450	100%

**Table 11:** APGAR score at 1 min

**APGAR Score @ 1min**	**Hyperthyroidism**		**Hypothyroidism**		**Normal**		**Total**	
	**Cases**	**%**	**Cases**	**%**	**Cases**	**%**	**Cases**	**%**
7 or more	2	100%	8	28.60%	328	78.10%	338	75.10%
4 to 6	0	0.00%	20	71.40%	64	15.20%	84	18.70%
< 4	0	0.00%	0	0.00%	28	6.70%	28	6.20%
Total	2	100%	28	100%	420	100%	450	100%

**Table 12:** APGAR score at 5 min

**APGAR Score @ 5min**	**Hyperthyroidism**		**Hypothyroidism**		**Normal**		**Total**	
	**Cases**	**%**	**Cases**	**%**	**Cases**	**%**	**Cases**	**%**
7 or more	2	100%	20	71.40%	364	86.70%	386	85.80%
4 to 6	0	0.00%	8	28.60%	32	7.60%	40	8.90%
< 4	0	0.00%	0	0.00%	24	5.70%	24	5.30%
Total	2	100%	28	100.00%	420	100%	450	100%

**Table 13:** NICU admission & TD

**NICU Admission**	**Hyperthyroidism**		**Hypothyroidism**		**Normal**		**Total**	
	**Cases**	**%**	**Cases**	**%**	**Cases**	**%**	**Cases**	**%**
NO	2	100%	20	71.40%	336	80.00%	358	79.60%
YES	0	0.00%	8	28.60%	84	20.00%	92	20.40%
Total	2	100%	28	100.00%	420	100%	450	100%

**Table 14:** treatment for TD

**Has mother received treatment for Thyroid disease?**	**Hyperthyroidism**		**Hypothyroidism**		**Total**	
	**Cases**	**%**	**Cases**	**%**	**Cases**	**%**	
Yes	2	100%	20	71.40%	22	73.30%
No	0	0.00%	8	28.60%	8	26.70%
Total	2	100%	28	100%	30	100%

**Table 15:** Complication (HT)

**Complications**	**Treatment received**		**Treatment not received**		**P value**
	**Cases**	**%**	**Cases**	**%**	
Present	6	28.60%	5	78.10%	< 0.01
Not present	14	71.40%	3	15.20%	
Total	20	100.00%	8	100.00%	
